# A Case Report of Parry–Romberg Syndrome Misdiagnosed as Multiple Sclerosis

**DOI:** 10.3389/fneur.2020.00797

**Published:** 2020-08-04

**Authors:** Ling Long, Zhuang Kang, Shaoqiong Chen, Chunping Cui, Xuejiao Men, Wei Qiu

**Affiliations:** ^1^Department of Neurology, The Third Affiliated Hospital of Sun Yat-sen University, Guangzhou, China; ^2^Department of Radiology, The Third Affiliated Hospital of Sun Yat-sen University, Guangzhou, China

**Keywords:** autoimmune, mechanism, Parry-Romberg syndrome, multiple sclerosis, MRI

## Abstract

**Background:** Parry–Romberg syndrome (PRS) is a rare disease that causes hemiatrophy of the face. The pathophysiological mechanisms involved in its etiology are unknown, but several previous reports suggest the involvement of autoimmune factors. Herein we describe the case of a patient with PRS who was initially misdiagnosed as having multiple sclerosis (MS). The relevant literature is briefly reviewed, and some previous reports suggesting associations between PRS and autoimmunity are discussed.

**Case Presentation:** A 34-year-old man presented with recurrent paroxysmal weakness of the right hand, a 3-years history of unilateral tinnitus, and headache for 6 months. MS was initially diagnosed but the patient was subsequently diagnosed as having PRS on the basis of clinical manifestations and radiological findings.

**Conclusions:** PRS may be associated with autoimmune pathogenesis, but the present case does not support that theory.

## Background

Parry–Romberg syndrome (PRS) is a rare disease characterized by progressive unilateral facial atrophy. Its etiology is not well-understood, but the involvement of autoimmunity has been suggested. Herein we report the case of a patient with severe white matter hyperintensity on T2 brain magnetic resonance imaging (MRI) who was initially misdiagnosed as having multiple sclerosis (MS). A short review of the literature pertaining to potential associations between PRS and autoimmunity is also provided.

## Case Presentation

A 34-year-old Chinese man presented with recurrent paroxysmal weakness of the right hand, tinnitus in the left ear for the past 3 years, and headache for the past 6 months. The patient said he had had exotropia of the left eye and left-sided hemi-facial atrophy since birth. His mother had reportedly suffered from tinnitus and hearing loss in her left ear from the age of 60 years, but no other potentially relevant family history was reported. He had been admitted to a local hospital 4 months earlier, and on that occasion brain MRI depicted diffuse lesions in the left cerebrum, resulting in a diagnosis of MS. He was treated with bolus methylprednisolone (1,000 mg/d for 5 days, two periods) followed by gradual transition to oral therapy, but did not respond to this therapy and was consequently referred to our center.

**Figure 1:**
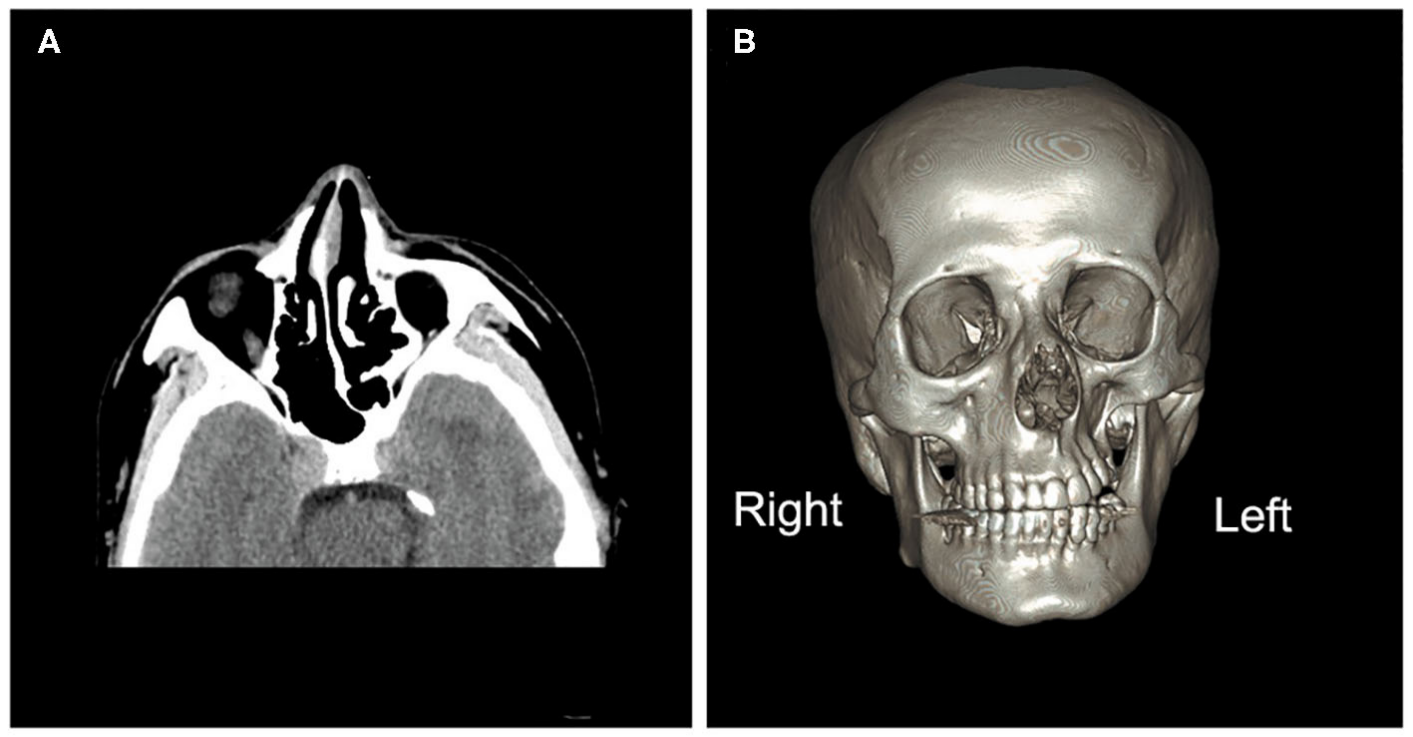
Brain computed tomography images. **(A)** Brain computed tomography (axial image) depicting under-development of the bone structure on the left side of the face compared with the right, including the nasal bone, nasal septum, sphenoid bones, and ethmoid bones. **(B)** A three-dimensional image of the brain depicting atrophy of the orbit, zygomatic bone, and teeth on the left side.

**Figure 2:**
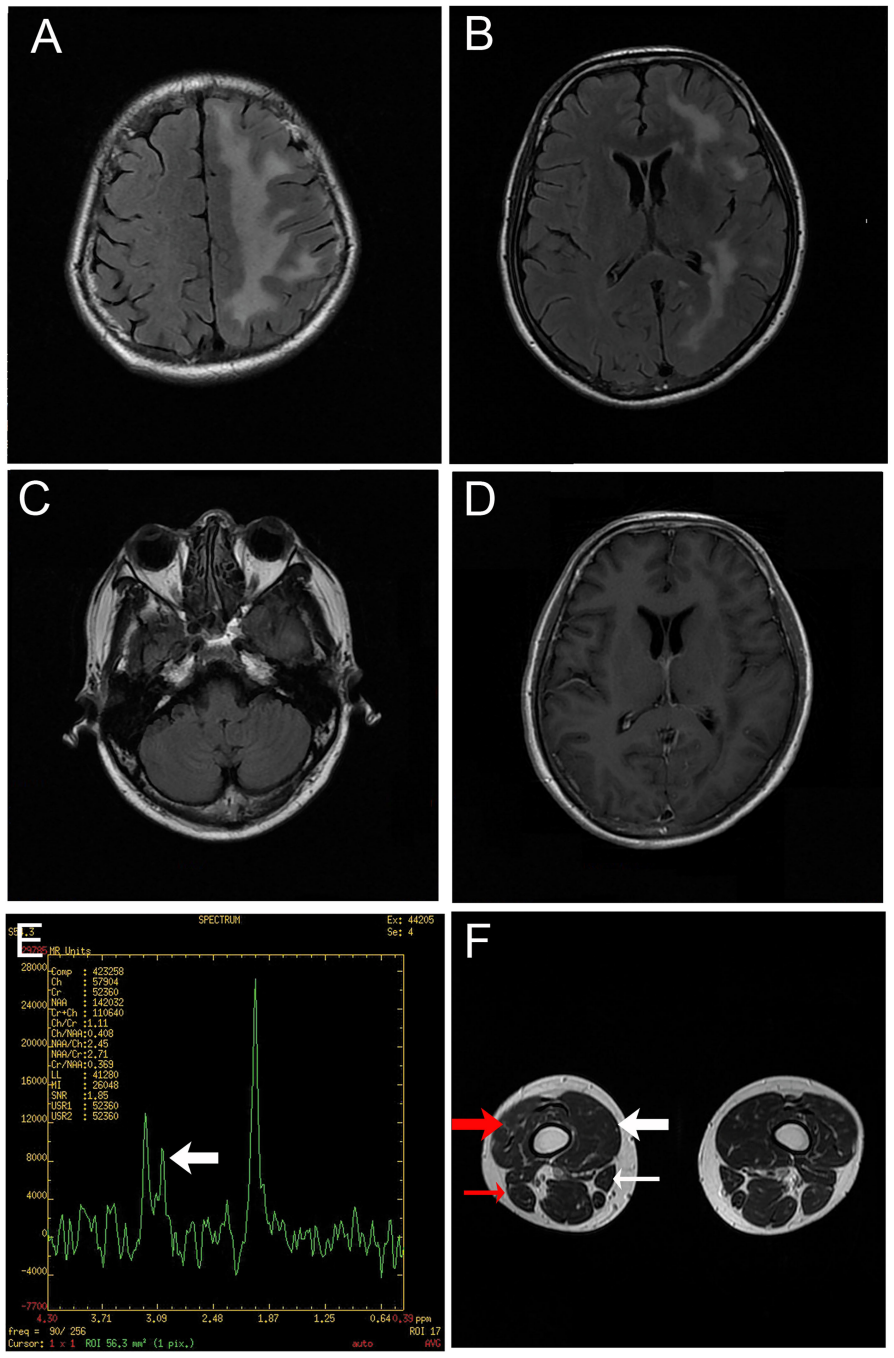
Magnetic resonance images. **(A**–**C)** T2-weighted fluid-attenuated inversion recovery images of the brain depicting diffuse hyperintensity signals in the left cerebrum. **(D)** Gadolinium enhanced T1-weighted image showing no enhancement of the lesion. **(E)** Hydrogen magnetic resonance spectroscopy image of a lesion in the left cerebrum, including a decreased choline peak (arrow). **(F)** Magnetic resonance image of the legs revealing muscular atrophy of the right leg, particularly the vastus medialis (thick white arrow), vastus intermedius (thick red arrow), sartorius (thin white arrow) and biceps femoris (thin red arrow).

Intracranial pressure was normal as determined via lumbar puncture examination, and the white blood cell count in cerebrospinal fluid (CSF) was 9^*^10e6/L. CSF total protein was 494 mg/L, CSF oligoclonal bands were negative, and the CSF autoimmune encephalitis antibodies NMDAR-IgG, AMPA1-IgG, AMPA2-IgG, LGI1-IgG, CASPR2-IgG, and GABA B-IgG were all negative. Autoimmune antibodies such as antinuclear antibody, anti-neutrophil cytoplasmic antibody, anticardiolipin antibody, anti-SSA, anti-SSB, and anti-double-stranded-DNA were negative. Aquaporin 4 (AQP4) antibody, myelin oligodendrocyte glycoprotein (MOG) antibody, and myelin basic protein antibody were negative. Neurological examination revealed left-sided hemi-facial atrophy and mild atrophy of the right lower limb. Hearing tests revealed hearing loss at high frequencies in the left ear. Three-dimensional computed tomography imaging of the brain depicted atrophied bone structure (Figure 1). MRI revealed diffuse lesions in the left cerebrum (Figures 2A–C), which were also evident in the previously conducted MRI. There was no enhancement of lesions in Gadolinium enhanced T1-weighted image (Figure 2D). Hydrogen magnetic resonance spectroscopy (H-MRS) image showed decreased choline peak of the lesion (Figure 2E). Additional MRI of the legs depicted muscular atrophy in the right leg (Figure 2F). The results of whole exome sequencing included INF2 (c.2410G>C) and GJB2 (c.109G>A). INF2 (c.2410G>C) has never been reported before, and the patient did not exhibit any evidence of related situation which had Charcot-Marie-Tooth disease with minimal glomerular dysfunction; thus, it was concluded that INF2 (c.2410G>C) was not pathogenic. GJB2 (c.109G>A) could explain the patient's tinnitus and hearing loss. The diffuse brain lesions required differentiation from neoplasms. There was no enhancement of the lesions, and CSF tests were all normal with no heteromorphic cells. On the basis of the clinical manifestations and radiological findings the patient was diagnosed as having PRS.

## Discussion and Conclusions

PRS is also known as progressive hemifacial atrophy, progressive facial hemiatrophy or Romberg's disease. Its incidence is estimated to be at least 1/700,000 (1). Symptoms usually occur in the first or second decade of life, and there is a female preponderance. Neurological symptoms such as epilepsy, headache, ipsilateral facial pain, facial deficits, dizziness, and tinnitus may be present in up to 15% of patients (2). Ophthalmic involvement is occasionally seen, with the most frequent form being enophthalmos (3). Computed tomography and MRI may reveal atrophy of subcutaneous tissues, fat, skin, underlying musculature, salivary glands and muscles, and/or deformed skeletal structure (4). Up to 74.6% of patients reportedly exhibit MRI-depicted abnormalities with T2 hyperintensity in the frontal lobe, parietal lobe, subcortical area, temporal lobe, occipital lobe or brain stem (5–7).

The present case is noteworthy for two main reasons. The first reason is that, in addition to the face the patient's contralateral lower limb was involved, a phenomenon that reportedly only occurs in 17.2% of patients (5). The other reason is that the brain MRI results were unique in that the lesions were widely distributed in the ipsilateral side to the atrophic face, indicating much greater severity than most previously reported cases.

The pathogenesis of PRS is not clear, but seems to be heterogeneous. Trauma is a cause of PRS in 24–34% of patients (8). Rarely, familial PRS has been reported (9), but no clear pattern of inheritance or evidence of one specific genetic defect has been found (10). Sympathetic nervous system dysfunction plays a key role in the pathogenesis of PRS, supported by the demonstration of clinical features of PRS after ablation of the superior cervical ganglion in animal studies (11). In some cases sympathectomy was sufficient to halt disease progression, suggesting that sympathetic irritation leads to facial hemiatrophy (12). Vascular abnormalities such as arteriovenous malformation and aneurysms are occasionally reported in PRS, supporting the hypothesis of failure of neural crest migration (13, 14). Proliferative interstitial neurovasculitis in histological examinations of facial specimens from patients with PRS (15) suggested that localized vasculitis leading to fragility of the arterial wall is another possible mechanism.

An increasing number of studies imply that autoimmune inflammatory processes play an important part in the development of PRS. It has been reported that there is a high incidence of autoimmune antibody positivity in patients with PRS. In a study by Garcia-de (16), 8/14 patients (57%) were positive for antinuclear antibody, and rheumatoid factor was detected in 5/14 (36%). Asanad (17) also described a patient with Hashimoto's thyroiditis who exhibited antinuclear antibody (1:40 speckled pattern).

In addition to reports implicating autoimmunity, there are indications that PRS can be evoked by infection. Zhang et al. (18) described a case of PRS that was apparently triggered by peripheral hepatitis B virus. Asanad (17) described a patient who developed PRS after herpes zoster infection, with post-herpetic neuralgia in the distribution of the ophthalmic division of the trigeminal nerve.

Some patients with PRS exhibit a “relapsing-remitting” course, which is a common feature of MS (18). Our patient suffered from recurrent paroxysmal weakness of the right hand, but the symptom occurred for about 1 h almost every day for 3 years. Therefore, the clinical course was not the “relapsing-remitting” type that is frequently observed in MS.

Brain MRI lesions in PRS are often located in similar areas to those observed in MS. In the present case, brain MRI depicted multiple white matter lesions in the frontal lobe, parieto-occipital area and periventricular areas, which are frequently seen in MS. It is likely that these lesions contributed to the initial misdiagnosis of MS in the current patient. However, there were no lesions located near the lateral ventricles, which are typical features of MS. Furthermore, the shapes of the lesions were not similar to those seen in MS. H-MRS images of the present case showed a decreased choline peak. The result of H-MRS of cases of PRS described in the literature are contradictory. Patients may present as normal (19, 20), or with decreased levels of Nacetylaspartate (NAA) and creatine together with the presence of lipid and lactate (21). Other cases have exhibited decreased NAA resonance together with increased choline resonance (5, 22). The changes observed with H-MRS reflect damage of brain tissues. The variety in the observed changes might be caused by variable degrees of damage and multiple mechanisms of pathogenesis in different patients.

Immunosuppressive therapy has achieved good results in some patients with PRS. In a case report, a 10-year-old girl presenting with seizures was diagnosed with PRS and the *en coup de saber* variant of linear scleroderma after receiving methylprednisolone at 1,000 mg weekly for 12 weeks (23). With transition to a prednisone taper and 25 mg weekly subcutaneous methotrexate with transition to oral therapy, she became asymptomatic and radiologically stable for 3 years. After stopping methotrexate for 2 months however, she experienced an event involving syncope rather than seizure, and an MRI conducted at that time revealed fresh microhemorrhages. This case in the literature in an example of PRS responding well to corticosteroid. There are also other reported cases of PRS with accompanying seizures that were improved or stabilized via corticosteroids, of which some of the patients had *en coup de saber* linear scleroderma while others did not (24–26).

Although much of the literature support the etiology of autoimmunity, the observation of the patient reported here seemed to be contradictory. The extensive white matter lesions in the current patient were initially considered to be immunological demyelinating lesions, however, they were not characteristic of MS in either shape or size. Also, the CSF was negative for oligoclonal bands, which provided more evidence of excluding MS. Furthermore, the patient did not exhibit a relapsing-remitting course and did not respond to large-dose methylprednisolone followed by oral cortical steroids. Therefore, our case should not be diagnosed as MS. Other central nervous system autoimmune diseases such as neuritis myelitis optica spectrum disorder (NMOSD), MOG antibody-associated inflammatory demyelinating disorders (MOG-IDDs) and tumefactive demyelinating lesions (TDLs) should also be considered. The patient did not meet the diagnostic criteria for NMOSD released by the International Panel for NMO Diagnosis (IPND) in 2015, so he could not be diagnosed with NMOSD. MOG-IDDs were excluded because MOG antibody was negative for this patient. Although pathological results were lacking, there was other evidence to exclude TDLs, including the long disease duration (>1 year) without deterioration and relatively mild symptoms, and no mass effect in brain MRI. It was therefore surmised that his condition was not likely to be any autoimmune disease that we have considered.

In conclusion, while immunological responses may contribute to the pathogenesis of PRS in many cases, numerous other aspects of the mechanisms potentially involved in the condition remain unknown.

## Data Availability Statement

The datasets generated and/or analyzed during the current study are available from the corresponding author on reasonable request.

## Ethics Statement

Written informed consent was obtained from the patient for the participation as well as the publication of the case report and any accompanying images. A copy of the written consent is available upon request from the corresponding author.

## Author Contributions

LL wrote the manuscript and was involved in the diagnostic and therapeutic clinical processes. ZK and SC contributed MR images and figure legends. CC contributed to the diagnostic and therapeutic processes. XM analyzed MR images. WQ analyzed the immunological aspects of patient data, helped in the diagnostic process and critically revising the manuscript, and was responsible for diagnosis and treatment of the patient. All authors read and approved the final manuscript.

## Conflict of Interest

The authors declare that the research was conducted in the absence of any commercial or financial relationships that could be construed as a potential conflict of interest.
